# Epidemiology, Clinical, Radiological and Biological Characteristics, and Outcomes of Mucormycosis: A Retrospective Study at a French University Hospital

**DOI:** 10.3390/jof10120884

**Published:** 2024-12-19

**Authors:** Tom Cartau, Sylvain Chantepie, Angélique Thuillier-Lecouf, Bénédicte Langlois, Julie Bonhomme

**Affiliations:** 1 Department of Parasitology-Mycology, CHU de CAEN Normandie, 14000 Caen, France; langlois-be@chu-caen.fr (B.L.); bonhomme-j@chu-caen.fr (J.B.); 2 Institute of Hematology of Basse-Normandie, CHU de CAEN Normandie, 14000 Caen, France; chantepie-s@chu-caen.fr; 3 Department of Nephrology, CHU de CAEN Normandie, 14000 Caen, France; thuillierlecouf-a@chu-caen.fr; 4 Institut National de la Santé et de la Recherche Médicale (INSERM) U1311 DYNAMICURE, Université de Normandie Unicaen, 14000 Caen, France; 5 ToxEMAC-ABTE, Université de Normandie Unicaen, 14000 Caen, France

**Keywords:** mucormycosis, Mucorales, infections, immunosuppressed, hematologic malignancies

## Abstract

Purpose: Mucormycosis is a rare but emerging and life-threatening infection caused by environmental mold, with a mortality rate of 30–70% despite progress in management. A better understanding could improve its management. Method: We conducted a single-center retrospective study of all cases of mucormycosis observed over a decade at the University Hospital of Caen. Results: Between 2014 and 2024, 18 cases of mucormycosis were identified, predominantly in males (n = 11, 65%). Most patients had hematological malignancies (n = 16, 89%). Seven cases were proven, and eleven were classified as probable. The main locations of infection were pulmonary (n = 12, 67%). Since 2021, we have observed an increase in the number of cases, rising from three between 2014 and 2021 to fifteen between 2021 and 2024. Among the 12 patients with pulmonary mucormycosis, all presented with fever except 1, and 67% required oxygen therapy. Chest computed tomography scans revealed an inverse halo sign in one-third of the patients. The first-line treatment consisted of amphotericin B in seventeen patients, posaconazole in one patient, and isavuconazole in one patient. Surgery was performed on seven patients. In cases of cutaneous mucormycosis, all patients underwent surgery, and none died within three months after the diagnosis. Overall, the three-month mortality rate was 39%. Surgical management was associated with a reduction in mortality (0% vs. 64%, *p* = 0.013). Conclusions: This study highlights the role of PCR for early diagnosis and the key role of surgery in improving clinical outcomes while underscoring the need for better-adapted therapeutic protocols for these rare infections.

## 1. Introduction

Mucormycosis is a rare but severe fungal infection caused by a group of environmental molds called Mucorales. Different species of Mucorales can lead to mucormycosis, the most common of which are *Rhizopus*, *Mucor*, and *Lichtheimia* [1]. Different clinical presentations are described, such as rhino-orbital-cerebral, cutaneous, pulmonary, or disseminated mucormycosis infections, depending on the underlying condition. Mucormycosis occurs mostly in diabetic or immunocompromised patients, such as those with hematological malignancies (HM) or solid organ transplantation (SOT). Cases have also been reported in immunocompetent patients or following a trauma or burns [2]. More recently, COVID-19–mucormycosis associations have been described, suggesting an underestimation of this disease [3]. Overall, various studies have reported a global increase in mucormycosis incidence [1,4,5]. Without a rapid diagnosis of the infection and aggressive treatment, this disease may be fatal. First-line treatment includes antifungal agents with high-dose amphotericin B and surgery, if possible, to limit fungal spread [6]. Even with optimal treatment, the mortality rate is approximately 30–70% [7]. Recently, a broader panel of antifungal agents has become available. Posaconazole is now recommended as an antimold prophylaxis in high-risk patients, such as those with HM, or for curative treatment of mucormycosis. Isavuconazole is now specifically indicated for the curative treatment of mucormycosis [8].

Mycological evidence for mucormycosis diagnosis is based on anatomopathological examination, direct examination, and fungal culture. New methods of Mucorales detection, such as PCR, have been developed and are used in France in current practice for mucormycosis diagnosis [9,10]. However, data are currently insufficient to globally recommend Mucorales PCR as mycological evidence for mucormycosis diagnosis in international recommendations, such as those of the 2019 European Organization for Research and Treatment of Cancer/Mycoses Study Group (EORTC/MSG) [11].

The incidence of mucormycosis is difficult to assess because of the low number of cases, but it is estimated to be 0.065/10,000 hospitalization days in France [4]. Mucormycosis is responsible for approximately 2% of invasive fungal infections (IFIs) in SOT patients and approximately 4–8% of IFIs in hematologic patients [12,13]. Given the low rate of mucormycosis, data on the global epidemiology, clinical and radiological presentation, treatment, and outcome of this disease are scarce. More recent studies have focused on the COVID-19–mucormycosis association [3,14], and a recent cohort has been published on the epidemiology and prognosis of mucormycosis in France [4]. However, data on the clinical, biological, and radiological features, outcomes, and prognoses of patients with mucormycosis are still limited.

The aim of this study was to describe the epidemiology, clinical and radiological characteristics, treatment, and outcomes of patients with mucormycosis in a French University Hospital over the last 10 years and to compare this local cohort with a recent national cohort [4].

## 2. Materials and Methods

We performed a single-center retrospective study including all cases of mucormycosis hospitalized at the University Hospital of Caen between January 2014 and June 2024.

The Caen University Hospital is the main hospital in the Lower Normandy region, France, and it comprises just over 1400 inpatient beds. It is the only hematology center in the region. In 2023, the hospital recorded approximately 135,000 hospital stays and performed 25,000 surgical procedures. Seventy-nine kidney transplants and ninety-five hematopoietic stem cell transplants were performed. No other types of transplants are performed at the facility. All surgical specialties are represented, as are all medical specialties, including oncology, which accounted for approximately 27,000 stays in 2023, and a pediatric oncology department. The hospital does not have a burn center.

Mucormycoses were classified as proven or probable according to the 2019 modified EORTC criteria [11]. Proven cases were defined by the association of host factors, clinical and radiological features compatible with mucormycosis, and one of the following mycological pieces of evidence: histopathological, cytopathologic, or direct microscopic examination of a specimen obtained by biopsy or positive culture of a specimen from a normally sterile site excluding bronchoalveolar lavage (BAL) fluid or urine.

Probable mucormycosis was defined by the association of host factors, clinical and radiological features compatible with mucormycosis, and the presence of Mucorales recovered by culture or by microscopic detection from sputum, BAL, bronchial brush or aspirate, or sinus aspirate samples.

Since 2021, Mucorales PCR has been available at our center to diagnose and screen patients at high risk of IFI, which has allowed us to add a biological argument for proven and probable cases. A real-time PCR kit was used to detect the ribosomal DNA of Mucorales without identifying the genus or species (Mycogenie^®^ *Aspergillus-Mucorales* spp., Ademtech, Pessac, France). PCR was included in our diagnosis algorithm as follows: two successive positive PCRs on blood samples or two simultaneously positive PCRs on different samples (blood and respiratory, for example) are mycological evidence for probable or proven mucormycosis, depending on the location of the sample. For high-risk patients with HMs, blood samples have been screened by PCR twice weekly since 2021. For other patients, blood PCR monitoring was not standardized.

Based on previous studies and the classification given in international guidelines, cases were identified as pulmonary mucormycosis, rhino-orbito-cerebral mucormycosis, cutaneous mucormycosis, or mucormycosis of other locations, depending on the infection site. We separated the underlying conditions into HM, SOT, burns, severe trauma, diabetes, and other [4,6] groups.

The date of diagnosis was considered the day of the first biological evidence of mucormycosis (PCR, culture, anatomopathological examination, or direct examination).

Demographic and clinical data, such as age, sex, underlying conditions, body mass index (BMI), history of diabetes, chronic respiratory insufficiency, current CMV reactivation, current coinfection (bacterial, viral, or fungal infection), date of onset of symptoms attributable to mucormycosis, and presence or absence of fever, were retrospectively collected. For pulmonary mucormycosis, we collected whether oxygen was needed. For the analysis of cutaneous mucormycosis, we collected samples for localization. Computed tomography (CT) scans of the chest, abdomen, sinus and/or brain were also analyzed if necessary.

For all patients, we collected the following biological data: hemoglobin, platelet, leukocyte, C-reactive protein (CRP), creatinine, bilirubin, aspartate aminotransferase, alanine aminotransferase, alkaline phosphatase, gamma-glutamyl transferase, albumin, and ferritin levels.

Other fungal biomarkers, including beta D glucans (Fungitell STAT^®^, Associates of Cape Cod, East Falmouth, MA, USA) and galactomannan antigens (Platelia Aspergillus Ag, Bio-Rad, Marnes-la-coquette, France), were detected in the blood. These markers were measured to assess a potential differential diagnosis; in the case of mucormycosis, both remained negative. Mucorales PCRs, direct examinations, and fungal cultures were performed on various clinical samples, and if possible, an anatomopathological examination was performed. For positive cultures, species were identified via mass spectrometry, using Filamentous Fungi (Bruker, Bremen, Germany) and MSI-2 (https://msi.happy-dev.fr, accessed on 17 December 2024) databases and confirmed by the National Reference Center for Invasive Mycoses and Antifungals (CNRMA, Institut Pasteur, Paris, France).

Data regarding the management and outcome of mucormycosis were compiled, including antifungal prophylaxis and curative treatment: presence of antifungal treatment (prescribed molecules, dosage, date of initiation, antifungal relay if applicable), possible surgical treatment, and date of death.

Mortality was analyzed up to three months after mucormycosis diagnosis. Death was attributed to mucormycosis if the clinical history was compatible with a direct link between the actual episode of mucormycosis infection and death. Death was considered not attributable to mucormycosis if another cause of death was recorded by clinicians.

### Statistical Analysis

Categorizable variables were analyzed via Fisher’s exact test. The medians of the quantitative variables were analyzed via the Mann–Whitney–Wilcoxon test. Associations were considered significant if the *p* value was <0.05.

## 3. Results

### 3.1. General Characteristics

Between 2014 and 2024, 18 cases of mucormycosis occurring in 17 patients were identified, the majority of whom were men (n = 11, 65%), with a median age of 64 years. The main underlying condition was HM (n = 16, 89%), including four hematopoietic stem cell transplants (HSCTs). All patients with HMs underwent intensive chemotherapy or were in the postallogeneic transplant period. None of the patients were in remission. Other underlying conditions included one SOT (n = 1, 5.5%) and one other condition (n = 1, 5.5%).

Seven cases were classified as proven mucormycosis, and eleven were classified as probable mucormycosis.

The clinical presentations were mainly pulmonary presentations (n = 12, 67%), observed in 11 patients with HMs and 1 patient with SOT, followed by cutaneous presentations (n = 5, 28%) occurring in patients with HM and finally, a renal presentation (n = 1, 5%) occurring in a patient without identified underlying conditions. Details on clinical data for each patient are shown in Supplementary Materials Table S1. Analyses of biological parameters, expressed as medians [IQ1–IQ3], revealed low hemoglobin levels in all patients (8.3 [7.6–8.7] g/dL) and low platelet counts in all but one patient (28 [18–55] G/L). Neutrophil counts were below the normal range in 16 out of 18 patients (0 [0–0.4] G/L), and CRP was elevated in all but 2 patients (106 [28–270] mg/L). Notably, ferritin levels were above the upper limit in 11 out of 12 patients when available (2668 [1168–5922] ng/mL). The clinical characteristics of the patients are presented in Table 1. The treatments and outcomes are presented in Table 2.

**Figure 1:**
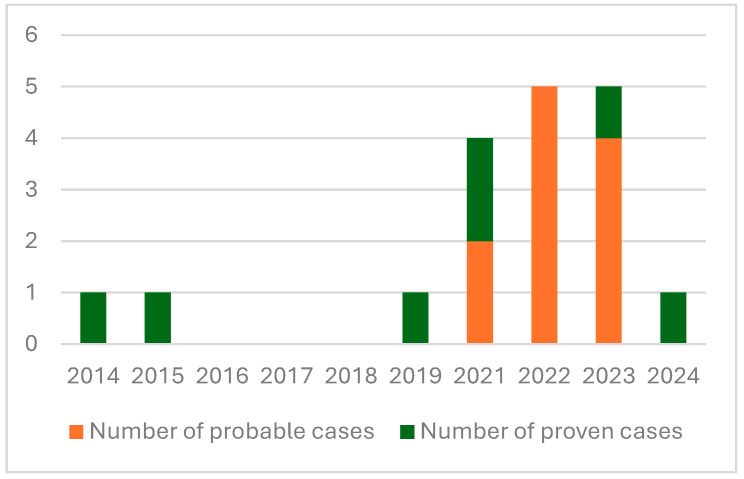
Evolution of the number of probable and proven cases of mucormycosis between 2014 and 2024 diagnosed at the University Hospital of Caen.

The distribution of cases over time is reported in Figure 1. The number of cases increased from three cases between 2014 and 2020 to fifteen cases between 2021 and 2024. The proportion of probable cases increased from 0/3 between 2014 and 2019 to 2/4 in 2021, 5/5 in 2022, 4/5 in 2023 and 0/1 in 2024.

For pulmonary mucormycosis (n = 12), fever was present in all patients except 1, and 5/12 presented with cough. Eight patients needed oxygen therapy. One case was associated with SARS-CoV-2 infection in a patient with SOT, one with adenovirus infection, and one with flu infection, both of which were in hematological patients. A chest CT scan revealed condensation in 12/12 patients and nodule condensation in 11/12 patients. A “reversed halo sign” was found in 4/12 patients, whereas a “halo sign” was found in 6/12 patients. Pleural effusion was found in 4/12 patients. Chest abnormalities were bilateral in 7/12 patients. The results of the CT scan are shown in Supplementary Materials Photographs S1. BAL was performed on 12 patients with suspected pulmonary mucormycosis. Direct examination revealed Mucorales in 2/12 patients, Mucorales PCR was positive in 7/11 patients, culture was positive in one patient, and anatomopathological examination revealed molds compatible with Mucorales in one patient. Blood PCR was positive in 9/11 patients when available.

For cutaneous mucormycosis (n = 5), lesions were localized to the face in one patient, the back in one patient, the arm in one patient, and the lower body in two patients. Photographs of lesions from two patients are accessible in Supplementary Materials Photographs S2. Fever was present in all patients. Biopsies were performed for all patients with cutaneous mucormycosis. Direct examination was always negative, culture was positive in 4/5 cases, anatomopathological examination revealed molds compatible with Mucorales in 2/3 cases, and PCR was positive in 4/5 cases. Blood PCR was positive in 2/3 of the patients when available.

With respect to renal mucormycosis (n = 1), direct examination and culture of urine were positive for two samples. Blood mucormycosis PCR was positive.

### 3.2. Mycological Evidence

Mucorales PCR was performed on the blood of 15/18 patients and was positive in 12 patients. It was the earliest positive mycological marker in eight cases. Blood PCR was performed in eleven patients with pulmonary mucormycosis, nine of whom were positive. PCR results were positive in two of three patients with cutaneous mucormycosis and two patients with renal mucormycosis.

For proven mucormycosis (n = 7), including five cases of cutaneous mucormycosis and two cases of pulmonary mucormycosis, blood PCR was positive in three cases, negative in one case, and not performed in three cases. Mycological criteria included direct examination and positive anatomopathological examination of surgical lung samples in one patient, positive anatomopathological examination of surgical skin samples in one patient, positive direct examination and culture of surgical skin samples in three patients, and positive direct examination of one surgical skin sample and one surgical lung sample.

For probable mucormycosis (n = 11), including 10 cases of pulmonary mucormycosis and one case of renal mucormycosis, all but two had positive Mucorales PCR results in the blood. One patient had positive direct examination, culture, PCR, and anatomopathological examination results on BAL in addition to positive blood PCR results. One patient had a positive direct examination and PCR on BAL fluid only. In four patients, PCR was positive in the blood and BAL fluid. In one patient, PCR was positive in the blood and in the pleural effusion. In one patient, PCR was positive in the blood, whereas culture and direct examination were positive in the urine. For three patients, at least two positive PCRs on blood samples were the only mycological evidence of mucormycosis.

The involved species were identified in six cases. *Lichtheimia ramosa* was isolated from three cutaneous biopsies, and *Rhizomucor pucillus* was isolated from another sample. *Rhizopus microsporus* was involved in two cases: one in a urine sample and one in a sputum sample. For other cases, a mycological diagnosis was obtained via PCR (n = 11) or direct examination (n = 1) without species identification.

### 3.3. Management and Outcomes

Twelve patients received antifungal prophylaxis, seven of whom were on posaconazole, which is effective against Mucorales. The others received antifungals that are not effective prophylaxis against Mucorales: voriconazole for one patient and fluconazole for four patients. Among the patients receiving posaconazole, blood concentration was measured in three, one of which was below the efficacy target (expected target ≥ 0.7 mg/L, blood level of posaconazole of three patients at 1.1 mg/L, 1.1 mg/L, and 0.6 mg/L, respectively). The reasons for the lack of antifungal prophylaxis are unknown.

The median time between the first clinical sign and treatment initiation was eight days [5–12.75], and the mean time was ten days.

The initial treatment was liposomal amphotericin B (LAmB) alone for sixteen patients with mucormycosis, the combination of LAmB and posaconazole for one patient (with pulmonary mucormycosis), and isavuconazole alone for one patient. LAmB was used at 4–5 mg/kg/day in 10/17 patients and 10 mg/kg/day in 7/17 patients. Patients developed adverse renal (2/18) or hepatic (9/18) effects in response to antifungal treatment. Only one patient who presented with pulmonary mucormycosis experienced relapse after treatment with posaconazole. Relapse occurred eight months after the first episode. He was managed by combination therapy with LAmB and posaconazole relayed by posaconazole. The patient was still alive without relapse after 6 months of follow-up.

Seven patients underwent surgical management: five patients had complete excision of a cutaneous lesion, one patient had an upper right lobectomy, and another had an upper right and partial middle lobectomy. All the patients who underwent surgery were alive at three months.

For patients with positive Mucorales PCR results in the blood (n = 12), PCR results in the blood became negative after a median duration of antifungal treatment of 11 days [6–15]. Blood PCR results were positive for a median of five days [1–14]. The persistence of Mucorales PCR positivity in the blood seven days after diagnosis was not associated with an increase in mortality at three months (50% vs. 50%, *p* = 1.0).

The overall mortality at three months after mucormycosis diagnosis was 39%. The mortality attributable to mucormycosis was 28%. The three-month mortality of pulmonary mucormycosis was 50%, whereas the mortality of cutaneous mucormycosis was 0%. The patient with renal mucormycosis died within three months. The median time from diagnosis to death was 32 days [3–99.5], and the mean time was 73 days.

The percentages of mortality at 3 months were not significantly different for patients with or without coinfections (45% vs. 28.5%, *p* = 0.64), pulmonary or other localization (50% vs. 17%, *p* = 0.32), cutaneous or other localization (0% vs. 54%, *p* = 0.1), presence of a positive Mucorales PCR in blood or not (50% vs. 17%, *p* = 0.32), persistence of Mucorales PCR in blood at day 7 or not (50% vs. 50%, *p* = 1).

## 4. Discussion

In this retrospective study, we described the epidemiology, clinical features, and outcomes of a case series of mucormycosis at a university hospital over a 10-year period. This study provided comprehensive clinical, radiological, and microbiological data on 18 patients with mucormycosis treated at a medium-sized French hospital.

Patients with HMs represented the majority of mucormycosis cases, and the proportion of these patients was greater than that reported in previous studies [4]. This difference could be explained by the fact that among SOT patients, only kidney transplants were performed at Caen University Hospital. Furthermore, the hospital did not have a specialized burn center.

The number of cases increased between 2014 and 2024, particularly after 2021, which is consistent with global epidemiological trends. The proportion of probable mucormycosis cases has also increased, likely due to the introduction of Mucorales PCR testing at our center in 2021. This PCR test is used not only to diagnose mucormycosis but also to screen patients at high risk of IFI, particularly those with HMs. Although Mucorales PCR is not yet recommended as a definitive diagnostic tool for mucormycosis, strong data now support its performance and value for early diagnosis [9,10]. The use of Mucorales PCR is widespread in France. In our study, Mucorales PCR was positive in blood samples from 12 of the 15 patients who underwent this test, and it provided the earliest mycological evidence in eight patients. Mucorales PCR on blood samples was the only mycological evidence for three patients with probable pulmonary mucormycosis. Mucorales PCR on blood and BAL samples identified infection in four other patients, whereas Mucorales PCR on blood and urine samples identified infection in one patient. Without the use of Mucorales PCR, these patients would not have been diagnosed. Only two out of eleven cases would be classified as probable mucormycosis without the use of Mucorales PCR. These findings underscore the importance of screening high-risk patients for suspected mucormycosis and guiding further diagnostic investigations. Blood PCR offers a valuable diagnostic tool, especially for patients unable to undergo BAL or surgery due to thrombocytopenia or severe respiratory disease.

Among the involved species, *Lichtheimia ramosa* was identified in three cases of cutaneous mucormycosis, which supports previous studies that showed that this species was more commonly associated with cutaneous forms of mucormycosis [15]. One case of cutaneous mucormycosis caused by *Rhizomucor pusillus* was identified from a surgical skin sample. This association is rare, with no reported cases of cutaneous mucormycosis caused by *Rhizomucor pusillus* reported in a recent national cohort [4], and to the best of our knowledge, only two cases of cutaneous mucormycosis in hematologic patients caused by *Rhizomucor pusillus* have been previously described [16,17]. The majority of species remained unidentified because the PCR used in Caen did not allow species identification. However, species identification is primarily of epidemiological interest and has no impact on the management of patients with mucormycosis.

The localization of mucormycosis significantly differed between probable and proven cases. The rate of cutaneous mucormycosis was significantly greater in proven cases than in probable cases (71% vs. 0%, *p* = 0.002), likely due to the ease of obtaining biopsy or surgical samples from patients with cutaneous forms of the disease. Pulmonary mucormycosis was significantly more common in probable cases (29% vs. 91%, *p* = 0.01), possibly reflecting the lower rate of surgical management and, consequently, fewer fungal and histopathological examinations. Many pulmonary mucormycosis cases were classified as probable based on positive Mucorales PCR results in the blood or BAL fluid, along with compatible clinical and radiological findings. However, these criteria are not yet accepted as mycological evidence for probable mucormycosis. Regarding the diagnostic performance and support provided by PCR, an update to international guidelines regarding the classification of mucormycosis would appear to be of interest.

For biological blood parameters, the elevated ferritin levels observed could indicate inflammation or macrophage activation syndrome, which reflects the intense inflammatory response triggered by Mucorales infections, as previously described [18]. Additionally, iron uptake is essential for Mucorales metabolism, and iron levels are correlated with the severity of mucormycosis [19,20,21]. However, data to support ferritin levels as a prognostic factor or an indicator for mucormycosis diagnosis are insufficient [22]. Thus, additional studies are needed to evaluate this prognostic factor.

With respect to imaging, the classic “reverse halo sign” was observed in only one-third of the patients, and the “halo sign”, which is more commonly associated with invasive aspergillosis, was observed in half of the patients. Diagnostic hypotheses are critical for guiding antimicrobial therapy in immunocompromised patients, and obtaining microbiological documentation is crucial. However, BAL is often contraindicated in patients with thrombocytopenia or severe respiratory symptoms. Because CT scan findings are nonspecific, we propose that any signs suggestive of fungal infection in high-risk patients should prompt the initiation of antifungal therapy active against Mucorales without waiting for a definitive diagnosis [23,24].

Regarding the initial treatment, LAmB was prescribed to 17 out of 18 patients, as recommended by international guidelines. The patient treated with isavuconazole received this treatment after its introduction as an alternative in the international guidelines.

The patient who relapsed was on maintenance therapy for eight months at the time of the new episode. In the absence of species identification, definitively concluding whether this represents a relapse or a new infection is impossible. However, regardless of the scenario, we believe that this episode can be analyzed independently, particularly in terms of biological parameters, imaging, and management, as described in this study.

The only factor significantly associated with three-month mortality was surgical management, which is consistent with the findings of previous studies [25]. Furthermore, we were unable to compare mortality rates based on underlying conditions because of the high proportion of hematologic patients.

Mortality was significantly greater among patients with probable mucormycosis than among patients with proven cases of the disease (0% vs. 64%, *p* = 0.013). This difference may be explained by the greater proportion of cutaneous involvement in proven cases and pulmonary involvement in probable cases. Moreover, proven cases were more likely to undergo surgery, which is an established prognostic factor in our cohort and in previous studies [4,25,26]. Patients with better general conditions are likely to undergo surgery. This bias in the selection of patients who undergo surgical management could explain the better outcomes of surgical management.

We compared our findings with those of a recent French national cohort of 550 cases of mucormycosis based on a national surveillance network [4], which included 12 patients from our study (those treated between 2012 and 2022). The main comparisons are shown in Supplementary Materials Table S2. We observed a greater proportion of patients with HMs in our series, likely due to a center-related effect. In fact, only kidney transplants were performed at our center, and burn patients were not treated here. In terms of disease localization, we did not observe any cases of rhino-orbito-cerebral mucormycosis, which is consistent with the underlying conditions described in both cohorts. Pulmonary mucormycosis remained the most common clinical presentation in patients with HMs, both in the national cohort and in our study.

We also observed a higher rate of cutaneous mucormycosis in patients with HMs than in the national cohort (31% vs. 5.3%, *p* = 0.002). The underlying hypothesis could be related to environmental contamination, which is supported by the environmental fungal survey performed at our center: Mucorales were found on sheets in the hematology department (with exposure to heavy air fungal loads because of construction work), leading to an increase in patient protection measures. Nevertheless, patients hospitalized in sterile hematology units during the construction period received active prophylaxis against Mucorales via posaconazole. Mucorales PCR results were negative on blood from one patient, whereas two patients did not undergo this test, and disease dissemination remained unexplained. This finding suggested direct contact contamination rather than hematogenous dissemination.

Management, antifungal therapy, or the rate of surgical intervention did not significantly differ between the cohorts. The 3-month mortality rate following a diagnosis of mucormycosis was lower in our study (38.9% vs. 55.8%), but this difference was not significant (*p* = 0.22).

## 5. Conclusions

This study confirmed that the increasing incidence of mucormycosis is linked to improvements in diagnostic tools, but poor prognosis persists. It also highlights the variability in clinical presentation, which depends on the underlying condition. Diagnosis remains challenging due to nonspecific clinical, radiological, and biological features. Mycological evidence is key to rapid diagnosis, and Mucorales PCR appears to improve diagnostic accuracy. Surgical management remains the primary prognostic factor. Further studies are needed to improve both the diagnosis and management of these patients, as well as to improve our understanding of the disease’s pathophysiology.

## Figures and Tables

**Table 1:** Baseline characteristics of 18 episodes of probable or proven mucormycosis diagnosed at the University Hospital of Caen between 2014 and 2024.

Baseline Characteristics	All Cases (n = 18)
Age, years, median [IQ1–IQ3]	64 [53–69]
Male, n (%)	12 (67%)
Underlying conditions, n (%)	
Hematologic malignancy	16 (89)
Receipt of HSCT	4 (22)
Graft versus Host reaction	2 (11)
SOT (kidney)	1 (5.5)
Other	1 (5.5)
Prolonged used of corticosteroids	2 (11)
Recent history of neutropenia	15 (83)
Diabetes	0 (0)
Localizations, n (%)	
Cutaneous	5 (28)
Lungs	12 (67)
Other	1 (5)
Species, n (%)	
*Lichtheimia ramosa*	3 (17)
*Rhizomucor pucillus*	1 (5)
*Rhizopus microsporus*	2 (11)
Unknown	12 (67)
For pulmonary mucormycosis, n (%)	12 (67)
Oxygen needed	8 (67)
Cough	5 (42)
Fever	11 (92)

HSCT: hematopoietic stem cell transplantation; SOT: solid organ transplantation.

**Table 2:** Antifungal prophylaxis received before the diagnosis of mucormycosis, first-line antifungal treatment against mucormycosis, and outcomes of 18 episodes of probable or proven mucormycosis diagnosed at the University Hospital of Caen between 2014 and 2024.

Characteristics	All Cases (n = 18)
Antifungal prophylaxis, n (%)	
Posaconazole	7 (39)
Fluconazole	4 (22)
Voriconazole	1 (5)
None	6 (33)
First line antifungal treatment, n (%)	
Liposomal amphotericin B	16 (89)
Isavuconazole	1 (5.5)
Liposomal amphotericin B + posaconazole	1 (5.5)
Surgical management	7 (39)
Mortality, n (%)	11 (61)
3-month mortality	7 (39)
3-month mortality attributable to mucormycosis	5 (28)

## Data Availability

The data that support the findings of this study are not openly available for reasons of sensitivity and data protection rules.

## Supplementary Materials

The following supporting information can be downloaded at: https://www.mdpi.com/article/10.3390/jof10120884/s1, Table S1: Main clinical characteristics of 18 cases of probable or proven mucormycosis between 2014 and 2024 at the University Hospital of Caen; Table S2: Comparison between our cohort and a national cohort in France, including mucormycosis cases between 2012 and 2022 [4]; Photographs S1: Chest CT-scan of twelve patients with probable or proven pulmonary mucormycosis; Photographs S2: Pictures of two patients with proven cutaneous mucormycosis before and after surgical management.
