# Supplementary material for: Epidemiology, Clinical, Radiological and Biological Characteristics, and Outcomes of Mucormycosis: A Retrospective Study at a French University Hospital
Source: J Fungi (Basel). 2024 Dec 19;10(12):884. doi: 10.3390/jof10120884 (PMC11677276; doi:10.3390/jof10120884)
Supplement: Supplementary file 1 [file jof-10-00884-s001.zip › jof-3321650-supplementary.pdf]

**Table S1: Main clinical characteristics of 18 cases of probable or proven mucormycosis between 2014 and 2024 at the University Hospital of Caen.**

| Diagnosis | Age | Sex | Underlying condition | BMI | Concomitant CMV reactivation | Bacterial or viral co-infection | Localization | Precision on cutaneous localization | Oxygen treatment | Cough | Fever | Antifungal prophylaxis | Surgical management | First line treatment | Overall three-month mortality |
|---|---|---|---|---|---|---|---|---|---|---|---|---|---|---|---|
| **Proven mucormycosis** | 27 | F | Hematologic malignancy | - | No | No | Lungs | - | Yes | No | Yes | Voriconazole | Yes | LAmB* | No |
| **Proven mucormycosis** | 61 | M | Hematologic malignancy | 27,3 | No | Yes | Cutaneous | Back | No | No | Yes | None | Yes | LAmB | No |
| **Proven mucormycosis** | 71 | F | Hematologic malignancy | 25,4 | No | No | Cutaneous | Seat | No | No | Yes | Fluconazole | Yes | LAmB | No |
| **Proven mucormycosis** | 65 | M | Hematologic malignancy | 27,8 | No | No | Cutaneous | Forarm | No | No | Yes | Posaconazole | Yes | LAmB | No |
| **Probable mucormycosis** | 77 | M | Hematologic malignancy | 24,8 | No | Yes | Lungs | - | No | Yes | Yes | Posaconazole | No | LAmB* | Yes |
| **Probable mucormycosis** | 68 | M | Hematologic malignancy | 28,3 | No | Yes | Lungs | - | Yes | Yes | Yes | Fluconazole | No | LAmB* | No |
| **Proven mucormycosis** | 15 | M | Hematologic malignancy | 15,9 | No | Yes | Lungs | - | No | No | Yes | None | Yes | LAmB* | No |
| **Probable mucormycosis** | 51 | M | HSCT | 29,2 | No | No | Lungs | - | No | No | Yes | None | No | LAmB* | Yes |
| **Probable mucormycosis** | 50 | M | Unknown | 26,9 | No | No | Kidney | - | No | No | Yes | None | No | Isavuconazole 600mg qd | Yes |
| **Probable mucormycosis** | 77 | F | SOT | 23 | Yes | Yes | Lungs | - | Yes | Yes | Yes | None | No | LAmB* | Yes |
| **Probable mucormycosis** | 73 | M | HSCT | 22,6 | No | Yes | Lungs | - | Yes | Yes | Yes | Posaconazole | No | LAmB* | No |
| **Probable mucormycosis** | 66 | F | Hematologic malignancy | 28,7 | No | Yes | Lungs | - | Yes | Yes | Yes | None | No | LAmB | Yes |
| **Proven mucormycosis** | 31 | M | HSCT | 29,2 | No | No | Cutaneous | Thigh | No | No | Yes | Fluconazole | Yes | LAmB | No |
| **Probable mucormycosis** | 59 | M | Hematologic malignancy | 22,9 | No | No | Lungs | - | Yes | No | Yes | Fluconazole | No | LAmB | No |
| **Probable mucormycosis** | 64 | F | Hematologic malignancy | 23,5 | No | Yes | Lungs | - | Yes | No | Yes | Posaconazole | No | LAmB | Yes |
| **Probable mucormycosis** | 59 | M | HSCT | 19,7 | No | Yes | Lungs | - | No | No | No | Posaconazole | No | LAmB* and Posaconazole 400mg qd | No |
| **Probable mucormycosis** | 70 | F | Hematologic malignancy | 28,5 | No | Yes | Lungs | - | Yes | No | Yes | Posaconazole | No | LAmB* | Yes |
| **Proven mucormycosis** | 66 | M | Hematologic malignancy | 27,3 | No | No | Cutaneous | Lips | No | No | Yes | Posaconazole | Yes | LAmB* | No |

*BMI: bone mass index; LAmB: liposomal amphotericin b at 10mg/kg daily; LAmB*: liposomal amphotericin B at 4-5mg/kg daily; HSCT: hematopoietic stem cells transplanted; SOT: solid organ transplanted.*

**Table S2: Comparison between our cohort and a national cohort in France, including mucormycosis cases between 2012 and 2022 [4].**

| | Our study, n=18 | Gouzien et al., n=550 | p-value |
|---|---|---|---|
| **Underlying conditions, n (%)** | | | |
| **Hematological patient** | 16 (89) | 358 (65) | **0,04** |
| **Solid organ transplanted** | 1 (5,5) | 36 (6,5) | 1 |
| **Burn or trauma** | 0 (0) | 73 (13,3) | 0,15 |
| **Localizations, n (%)** | | | |
| **Pulmonary mucormycosis** | 12 (67) | 288 (52,4) | 0,34 |
| **Cutaneous murcormycosis** | 5 (28) | 94 (17,1) | 0,22 |
| **Other mucormycosis** | 1 (5) | 27 (4,9) | 0,6 |
| **Rhino-orbito-cerebral mucormycosis** | 0 (0) | 80 (14,5) | 0,09 |
| **Classification of cases, n (%)** | | | |
| **Proven** | 7 (38,9) | 207 (37,7) | 1 |
| **Probable or putative** | 11 (61,1) | 343 (62,3) | 1 |
| **Outcome, n (%)** | | | |
| **3-month mortality** | 7 (38,9) | 276/495 (55,8) | 0,23 |

**Photographs S1: Chest CT-scan of twelve patients with probable or proven pulmonary mucormycosis.**

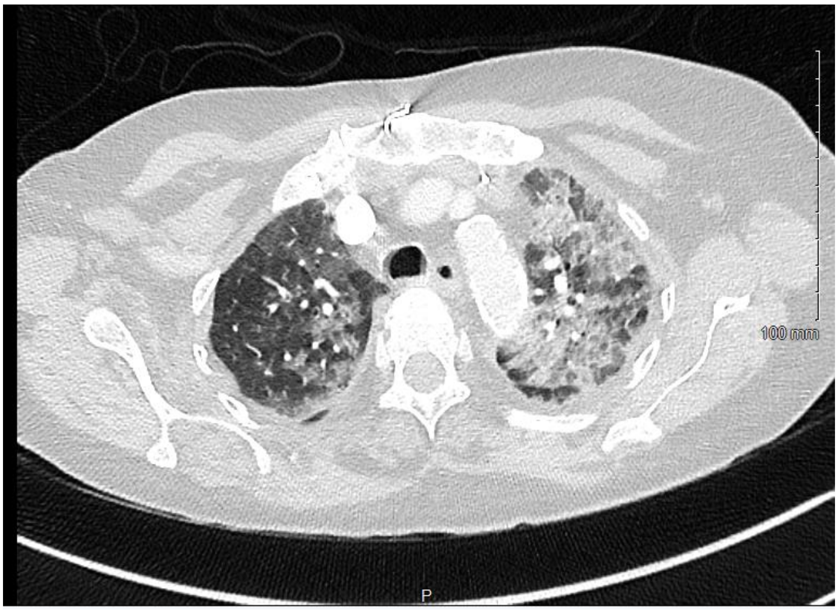


Figure S1. Patient with renal transplant and probable mucormycosis. Bilateral condensations and ground-glass condensations.

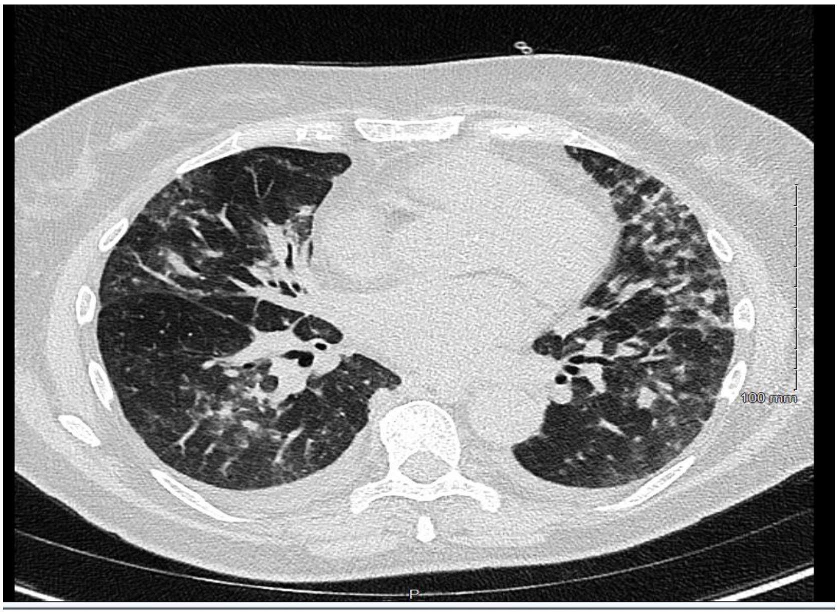


Figure S2. Patient with hematologic malignancy and probable pulmonary mucormycosis. Bilateral nodules and condensations.

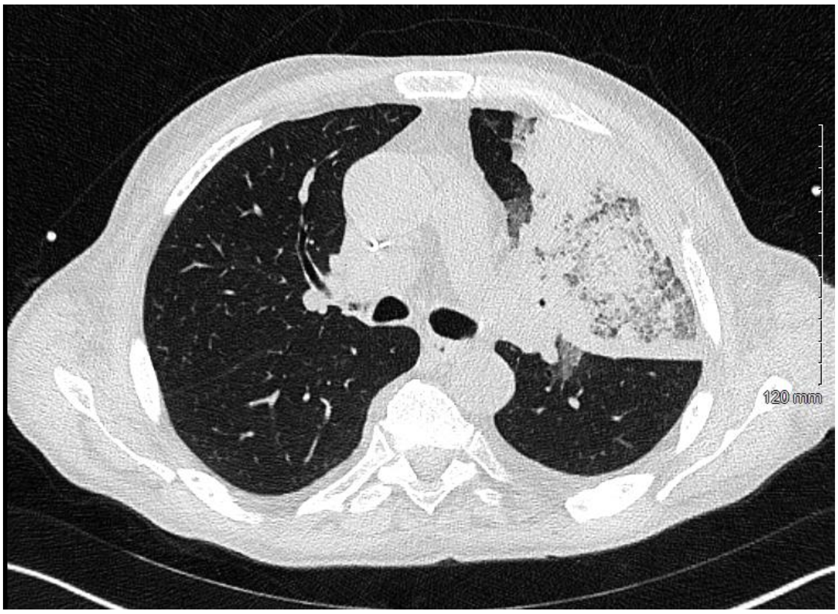


Figure S3. Patient with hematologic malignancy and probable mucormycosis. Reverse halo sign of the left upper lobe.

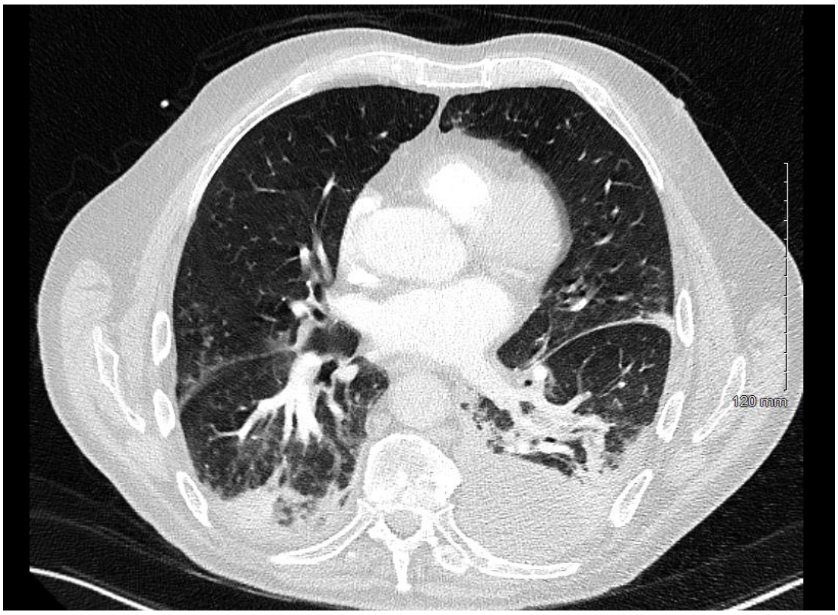


Figure S4. Patient with hematologic malignancy and probable mucormycosis. Bilateral condensation and a left septated pleural effusion.

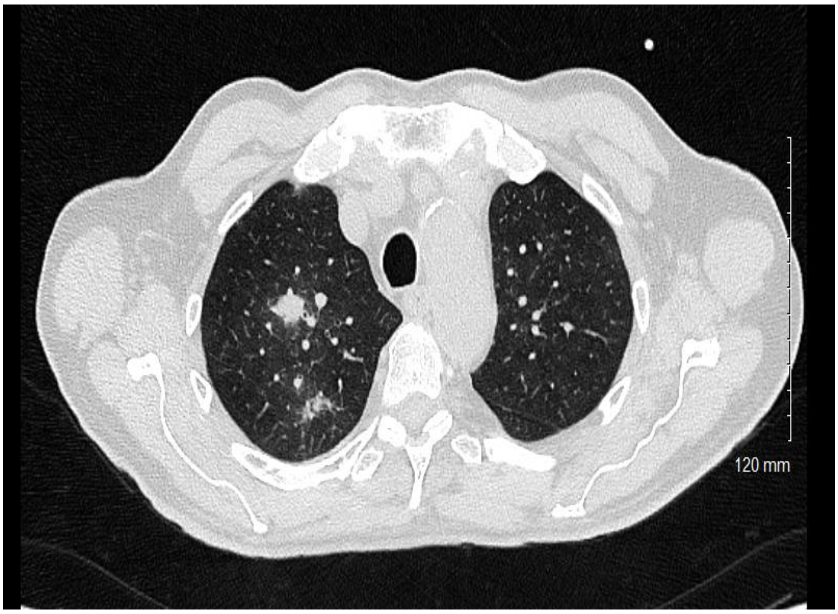


Figure S5. Patient with hematologic malignancy and a probable mucormycosis. Bilateral nodules.

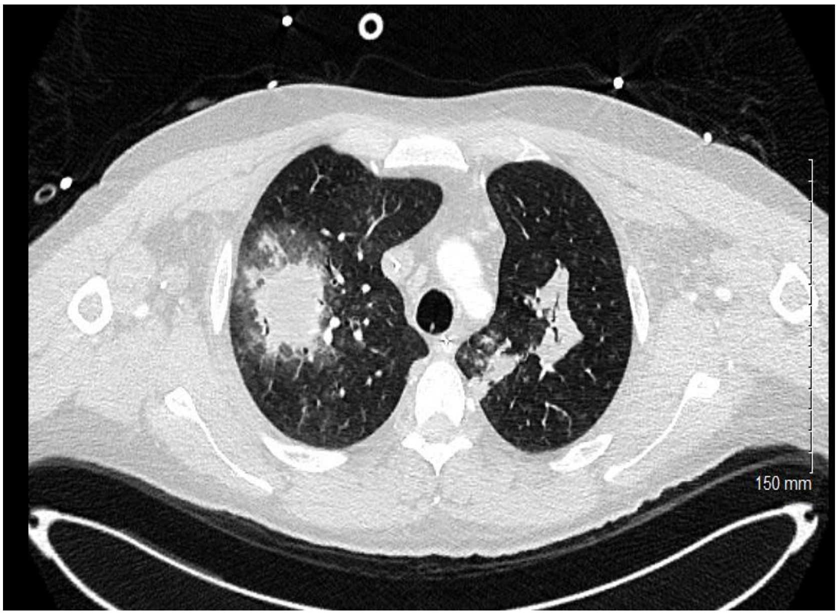


Figure S6. Patient with hematologic malignancy and probable mucormycosis. Bilateral condensations and a halo sign.

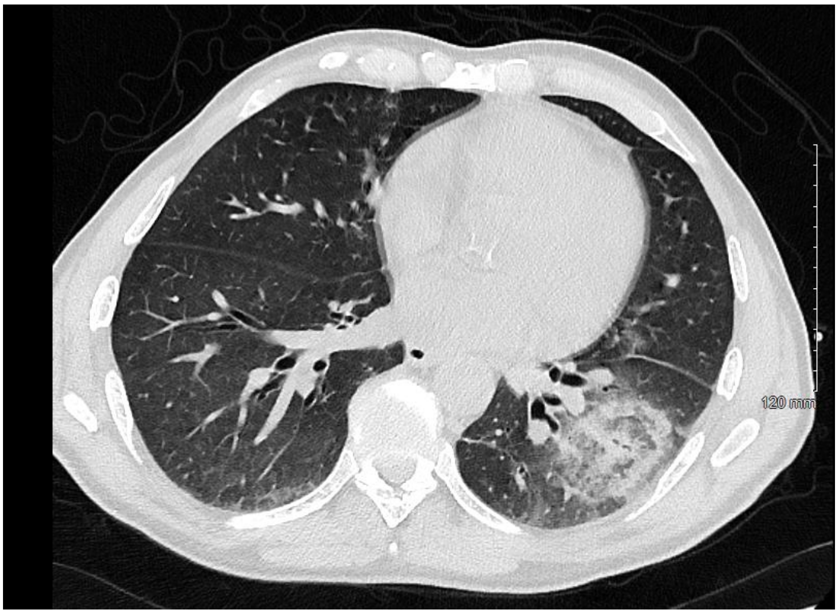


Figure S7. Patient with hematologic malignancy and probable mucormycosis. Condensation with a reversed halo sign of the left lower lobe.

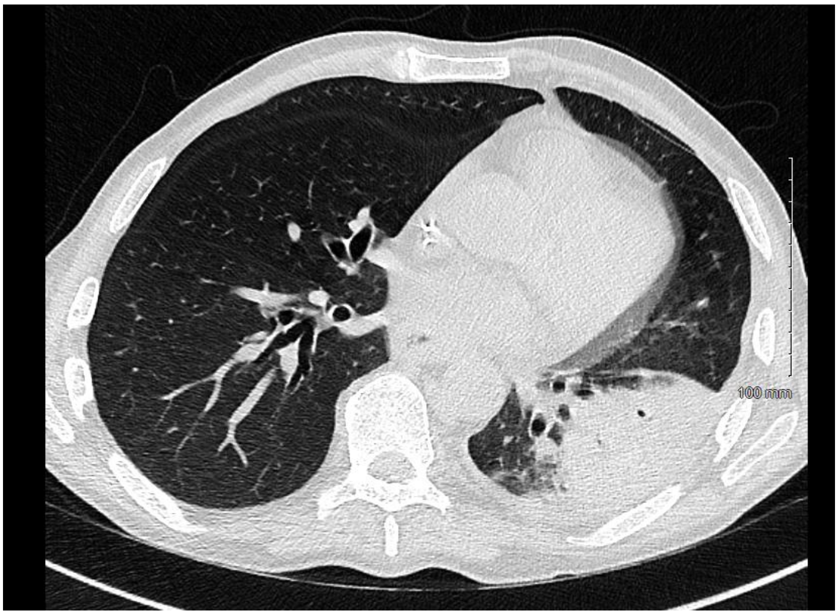


Figure S8. Patient with hematologic malignancy and probable mucormycosis (relapse of the case shown in Figure 7). Bilateral nodules and a condensation of the left lower lobe with signs of necrosis.

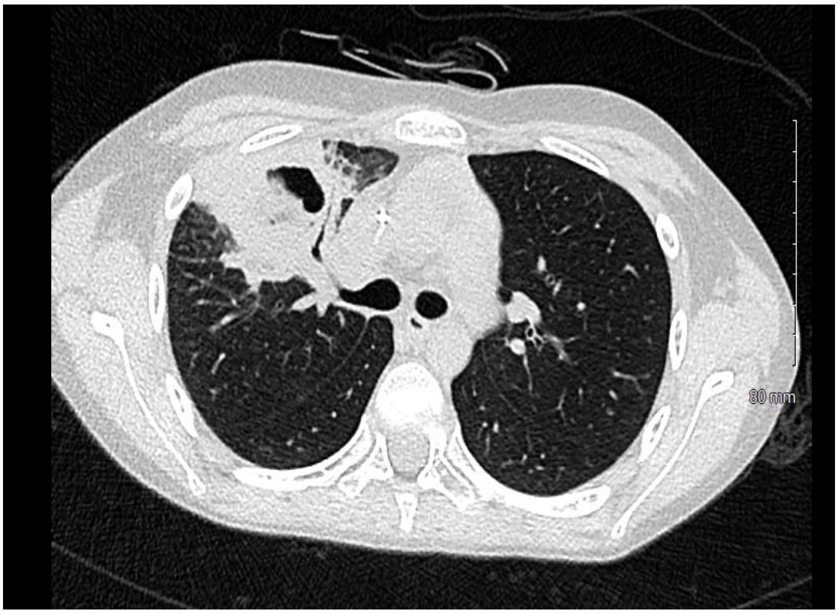


Figure S9. Patient with hematologic malignancy and proven mucormycosis. Necrotizing condensation of the right upper lobe and nodule of the left lung.

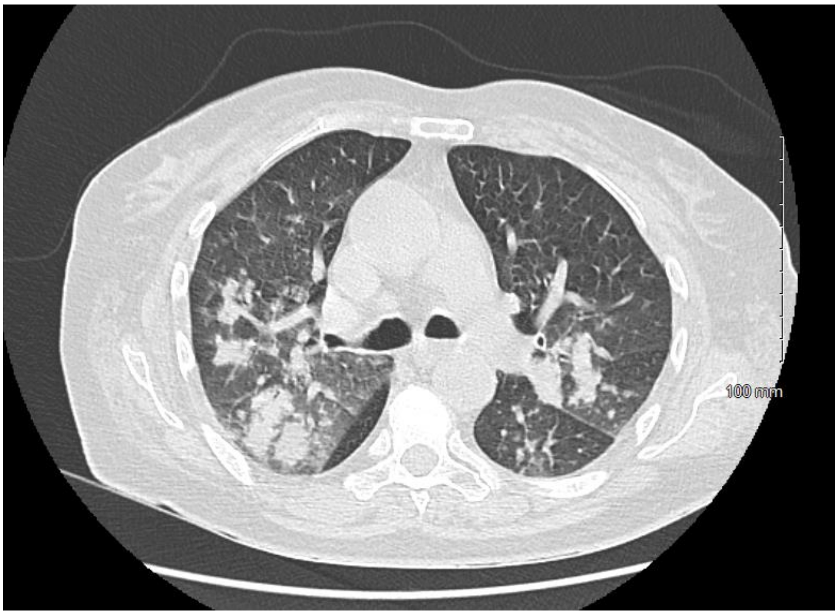


Figure S10. Patient with hematologic malignancy and probable mucormycosis. Bilateral condensations with halo sign and ground-glass condensations.

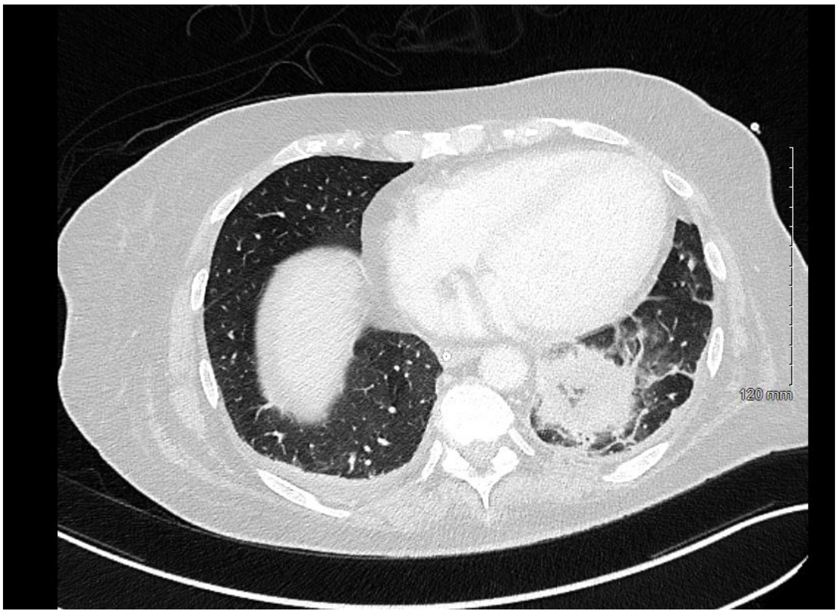


Figure S11. Patient with hematologic malignancy and probable mucormycosis. Condensation with a reversed halo sign.

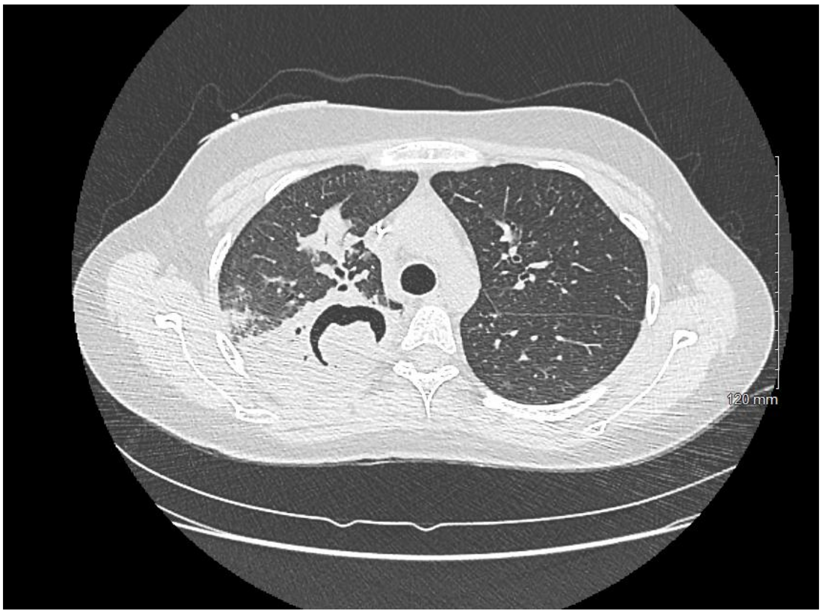


Figure S12. Patient with hematologic malignancy and proven mucormycosis. Necrotizing condensation of the right lower lobe.

**Photographs S2: Pictures of two patients with proven cutaneous mucormycosis before and after surgical management.**

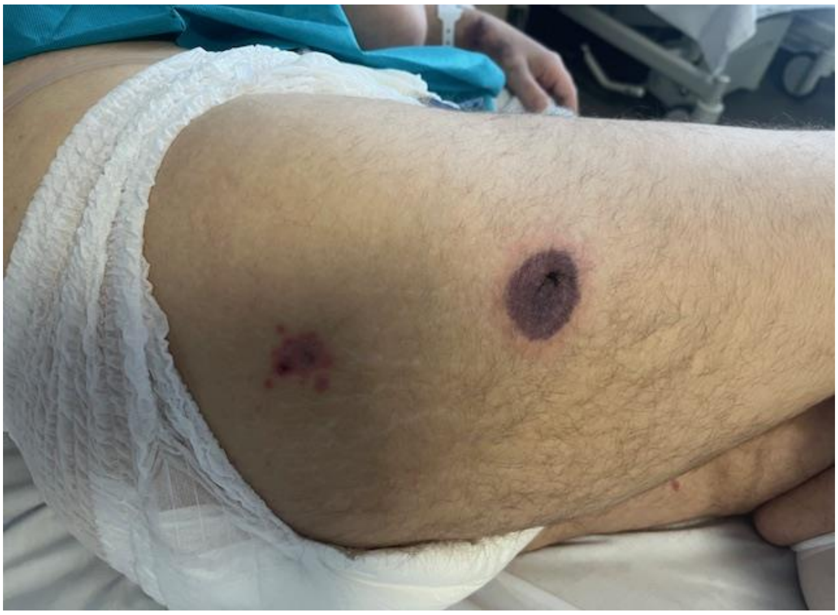

Figure S13. Necrotizing cutaneous lesion of the right thigh in a patient with hematologic malignancy.

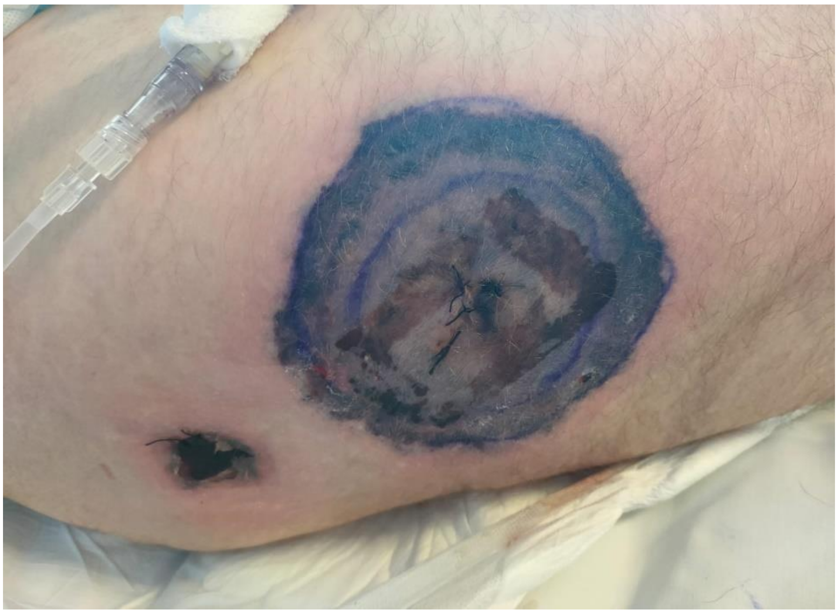

Figure S2. Necrotizing cutaneous lesion of the right thigh of the same patient shown in Figure 13, three days later.

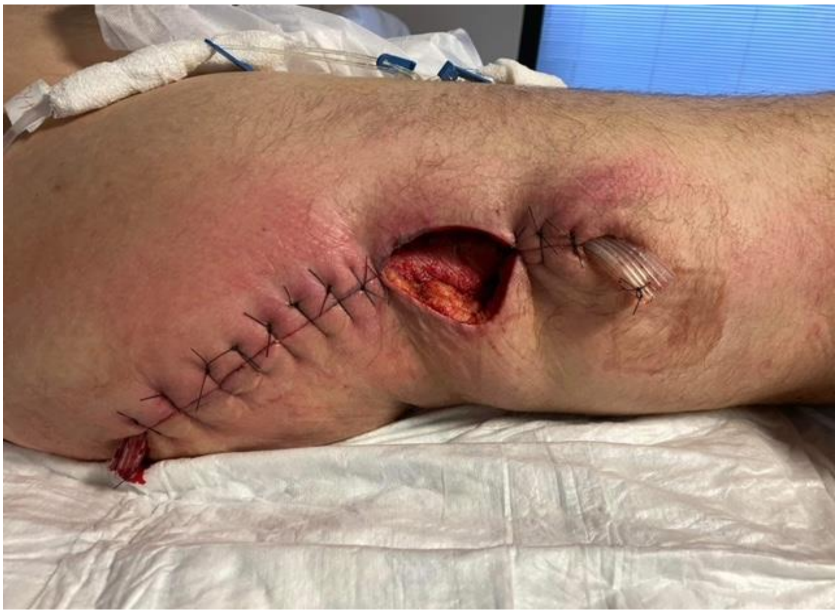

Figure S3. Post-surgical aspect of the right thigh of the same patient shown in Figure 1 and Figure 2. Figure 2 and Figure 3 were taken on the same day.

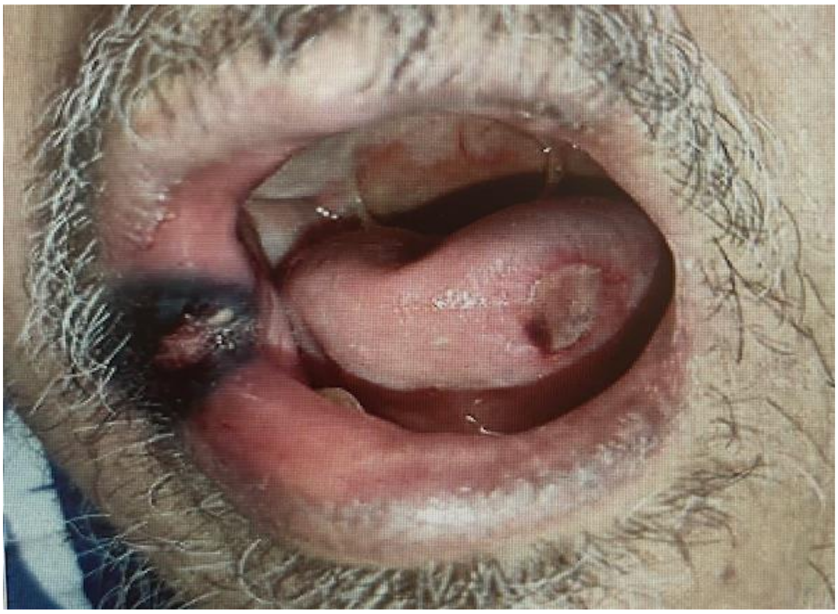

Figure S4. Necrotizing cutaneous lesion of the lip in a patient with hematologic malignancy.

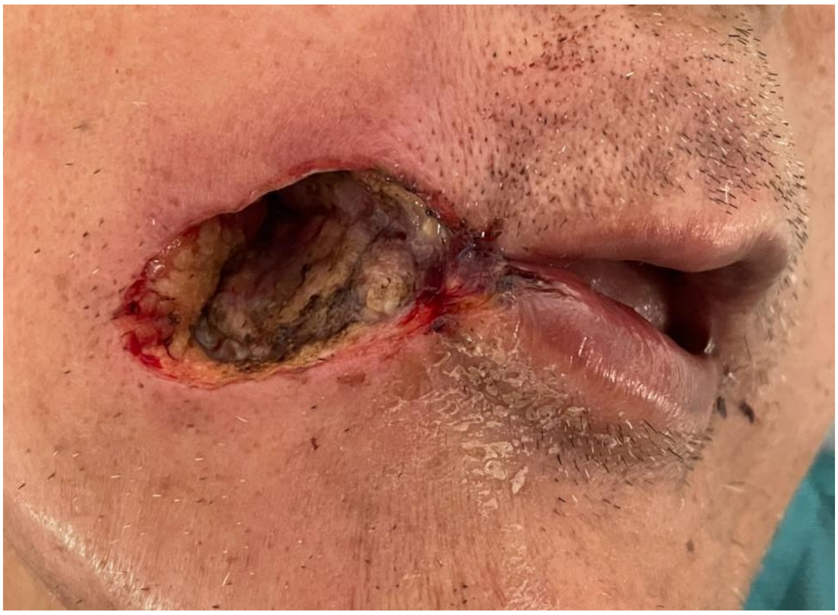

Figure S5. Post-surgical aspect of the same patient shown in Figure 16.

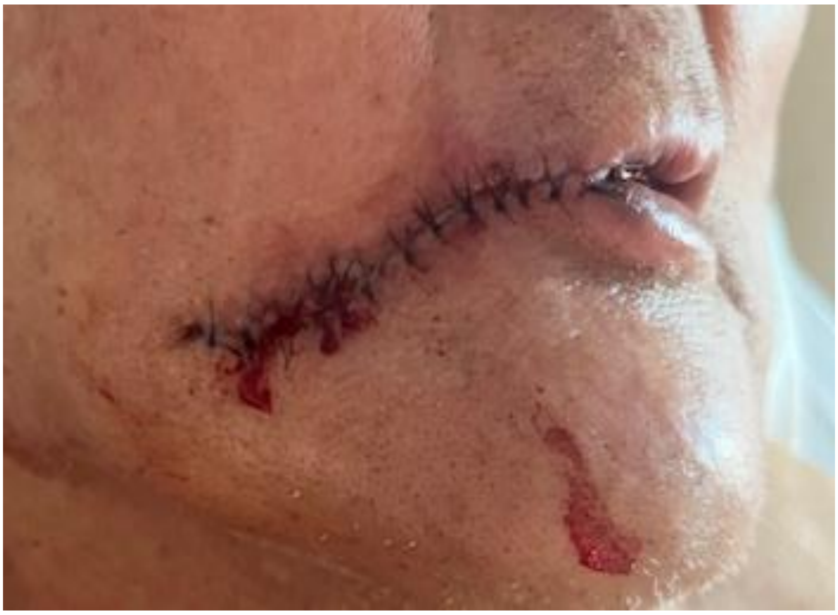

Figure S6. Aspect after surgical reconstruction of the same patient shown in Figure 4 and Figure 5. Reconstruction was performed three days after the first surgery.
